# The prior infection with SARS-CoV-2 study (PICOV) in nursing home residents and staff - study protocol description and presentation of preliminary findings on symptoms.

**DOI:** 10.1186/s13690-021-00715-z

**Published:** 2021-11-11

**Authors:** Maria E. Goossens, Kristof Y. Neven, Pieter Pannus, Cyril Barbezange, Isabelle Thomas, Steven Van Gucht, Katelijne Dierick, Marie-Noëlle Schmickler, Mathieu Verbrugghe, Nele Van Loon, Kevin K Ariën, Arnaud Marchant, Stanislas Goriely, Isabelle Desombere

**Affiliations:** 1 SD Epidemiology and Public Health, Sciensano, Brussels, Belgium; 2 SD Infectious Diseases in Humans, Sciensano, Brussels, Belgium; 3 Mensura Occupational Health Service, Antwerp, Belgium; 4 Virology Unit, Department of Biomedical Sciences, Institute of Tropical Medicine, Antwerp, Belgium; 5 Department of Biomedical Sciences, University of Antwerp, Antwerp, Belgium; 6 Institute for Medical Immunology, Université libre de Bruxelles, Charleroi, Belgium

**Keywords:** SARS-CoV-2, COVID-19, ILI, ARI, Multicentric, Cohort, Belgium, Antibody, Symptoms, Nursing home

## Abstract

**Background:**

The COVID-19 pandemic, caused by the severe acute respiratory syndrome coronavirus 2 (SARS-CoV-2), has presented itself as one of the most important health concerns of the 2020’s, and hit the geriatric population the hardest. The presence of co-morbidities and immune ageing in the elderly lead to an increased susceptibility to COVID-19, as is the case for other influenza-like illnesses (ILI) or acute respiratory tract infections (ARI). However, little is known, about the impact of a previous or current infection on the other in terms of susceptibility, immune response, and clinical course. The aim of the “Prior Infection with SARS-COV-2” (PICOV) study is to compare the time to occurrence of an ILI or ARI between participants with a confirmed past SARS-CoV-2 infection (previously infected) and those without a confirmed past infection (naïve) in residents and staff members of nursing homes. This paper describes the study design and population characteristics at baseline.

**Methods:**

In 26 Belgian nursing homes, all eligible residents and staff members were invited to participate, resulting in 1,226 participants. They were classified as naïve or previously infected based on the presence of detectable SARS-CoV-2 antibodies and/or a positive RT-qPCR result before participation in the study. Symptoms from a prior SARS-CoV-2 infection between March and August 2020 were compared between previously infected residents and staff members.

**Results:**

Infection naïve nursing home residents reported fewer symptoms than previously infected residents: on average 1.9 and 3.1 symptoms, respectively (p = 0.016). The same effect was observed for infection naïve staff members and previously infected staff members (3.1 and 6.1 symptoms, respectively; p <0.0001). Moreover, the antibody development after a SARS-CoV-2 infection differs between residents and staff members, as previously infected residents tend to have a higher rate of asymptomatic cases compared to previously infected staff members (20.5% compared to 12.4%; p <0.0001).

**Conclusions:**

We can postulate that COVID-19 disease development and symptomatology are different between a geriatric and younger population. Therefore, the occurrence and severity of a future ILI and/or ARI might vary from resident to staff.

## Background

At the end of 2019, a novel coronavirus causing acute respiratory disease (COVID-19) emerged in the Wuhan region of China and has since led to a worldwide pandemic. The causative agent was named ’severe acute respiratory syndrome coronavirus 2’ (SARS-CoV-2) and is closely related to SARS-CoV, which led to an epidemic between 2002 and 2004 [1]. It is estimated that around 40-45% of all SARS-CoV-2 infections remain asymptomatic [2], for which a dependency on age was observed [3]. Those who do develop COVID-19 present variable clinical outcomes, ranging from mild disease with typical symptoms including fever and cough to severe respiratory illness and death [4–7].

COVID-19 related mortality is strongly age-dependent, with the highest incidence of deaths reported in the geriatric population [6, 8, 9]. Comorbidities related to ageing as well as immunosenescence, i.e. decreased immunological competence due to biological ageing, lie at the basis of this increased risk of death with advanced age [10]. In Belgium, around 10% of the population aged 65 and older lives in nursing homes where they receive formal long-term care [11], and because of this high concentration of susceptible people in such facilities, more than 60% of all COVID-19 related deaths between March and June 2020 occurred in nursing home residents [12].

In addition, multiple studies have shown significant co-infection rates of SARS-CoV-2 infected patients with viruses causing influenza-like illnesses (ILI) or acute respiratory tract infections (ARI) such as seasonal mild corona viruses, influenza A/B virus, parainfluenzavirus, rhinovirus, bocavirus, human metapneumovirus, and adenovirus [13–16]. As is the case for COVID-19, geriatric people are more at risk for severe clinical forms of these infections. While there are indications that co-infection with e.g. influenza A might increase SARS-CoV-2 viral loads and thereby someone’s degree of contagiousness [17], in general little is known about the impact of one infection (previous or current) on the other in terms of susceptibility, immune response and clinical course.

We therefore conducted a prospective cohort study in Belgian nursing homes, including both residents and members of staff who were either previously infected with SARS-CoV-2 or not. Study participants were followed up during the 2020-2021 flu season by monitoring the incidence and severity of ILIs and ARIs, as well as determining their causal pathogen by multiplex molecular testing of naso-pharyngal swabs collected at the onset of symptoms. The primary objective of this multi-centric prospective cohort study was to assess whether a confirmed prior infection with SARS-CoV-2 (PICOV) affected the susceptibility to and severity of an ILI and ARI. The aim of the current paper is to describe the PICOV study design, sampling scheme, biological measurements as well as baseline population characteristics.

## Methods

### Study design

Over 200 nursing homes, located in all the Belgian regions of Flanders, Wallonia, and Brussels, were contacted and invited to participate in the multi-centre prospective PICOV study. Finally, 26 nursing homes agreed to participate. Both residents as well as staff were eligible to participate. We aimed to include an equal number of participants with and without a previous SARS-CoV-2 infection (i.e. naïve participants, and previously infected participants, respectively).

Participant were eligible when they were at least 18 years old, had a Belgian National Number, were insured by one of the Belgian sickness funds, and had the cognitive ability to give consent to participate. Participants with the following criteria were excluded from participating: participants who are unable to fill out questionnaires in Dutch or French, those whose life-expectancy is shorter than the duration of the study, those whose veins were inaccessible for a simple periphery venipuncture, those who will not work or reside at the nursing home for the full duration of the study, and those who have had a previous diagnosis of dementia or had a mini-mental state examination (MMSE) of 18/30 or less.

### Objectives of the PICOV study

The primary objective of the PICOV study is to compare the susceptibility to and the severity of an ILI and ARI in subjects who have had a confirmed prior infection with SARS-CoV-2 (i.e previously infected; detectable SARS-CoV-2 Ab at baseline and/or a positive SARS-CoV-2 RT-qPCR between March and August 2020) to those who have not experienced a SARS-CoV-2 infection (i.e. naïve; no detectable SARS-CoV-2 Ab at baseline and no positive SARS-CoV-2 RT-qPCR between March and August 2020). Specific study objectives, not investigated in the current paper, include:

- Determining which viruses are responsible for the ILI or ARI incidences throughout the study.
- Evaluating whether COVID-19 convalescent participants are protected against reinfection with SARS-CoV-2 and identifying serological markers that are associated with protection.
- Assessing the influence of a previous SARS-CoV-2 infection on the immunological response to influenza vaccination.

### Objectives of the current paper

The aim of the present manuscript is to describe the study design, sampling scheme, biological measurements, and population characteristics at baseline. Moreover, we investigate the differences in symptoms between naïve and previously infected staff members and residents.

### Study flow

#### Recruitment

Eligible staff members and residents of nursing homes, both naïve and previously infected, were invited to participate. We aimed to include a cohort of participants consisting of 50% infection naïve and 50% previously infected participants, in each nursing home. However, during the recruitment of participants into the study, previously asymptomatically infected people [18] and people with previous false-negative SARS-CoV-2 RT-qPCR test results [19] can be incorrectly classified as infection naïve, and can only be identified as previously infected after participation through serological testing. To account for this reality, we recruited 20% more participants who disclosed that they were infection naïve (based on previous test results only) than previously infected participants.

To also accommodate the inclusion of sufficient previously infected participants, a serological screening with an antibody rapid diagnostic test (RDT) was performed for people who never received a positive RT-qPCR or serological test. To better sustain selection of eligible people, this diagnostic screening only occurred in nursing homes where less than 50% of the staff members and residents have ever had a positive SARS-CoV-2 RT-qPCR test. Nursing homes where over 50% of the staff and residents were previously infected, enrolment commenced without screening. Antibody RDTs included the CORIS COVID-19 Ab Rapid Test (CORIS BioConcept, Gembloux, Belgium), COVID-PRESTO^®^ COVID-19 IgG/IgM Rapid Test (AAZ-LMB, Boulogne-Billancourt, France), and the quickZen COVID-19 IgG/IgM Rapid Test (Zentech, Liège, Belgium). Dates and results of previous lung CT-scans, RT-qPCR tests and/or antibody tests were collected by the study nurses to better classify potential participants. Final classification of participants as “naïve” or “previously infected” was done on the presence of detectable antibodies at baseline sampling and the result of any SARS-CoV-2 RT-qPCR test between March and August 2020.

#### Sample collection and biological measurements

**Fig. 1:**
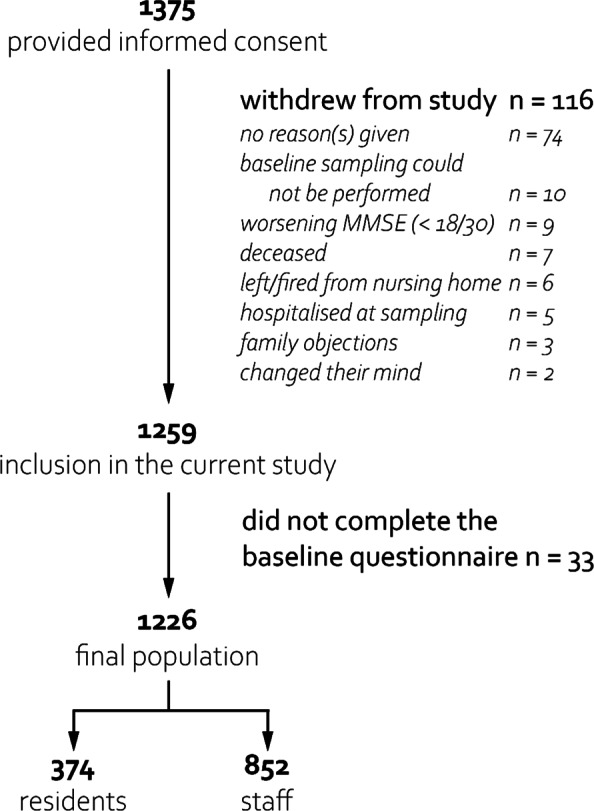
Flow of the PICOV study. Recruitment of the participants finished on December 8^th^, 2020, as did the baseline sampling (collection of a nasopharyngeal swab, saliva, serum blood, and heparinised blood for peripheral blood mononuclear cell [PBMC] isolation). Follow-up sampling after flu vaccination concluded on the 22^nd^ of January (collection of serum blood). Sampling at the end of the study is planned for spring 2021 (collection of saliva, serum blood, and heparinised blood for PBMC isolation). If a participant develops an influenza-like illness (ILI) or an acute respiratory infection (ARI), a nasopharyngeal swab is collected at the onset of symptoms to distinguish between a SARS-CoV-2 or influenza infection. After attenuations of the illness, approximately two to three weeks after the onset of symptoms, serum, and heparinised blood is collected

Biological samples, including nasopharyngeal swabs, saliva, and blood, were collected from all participants at baseline and at the end of the study. Figure 1 summarises which samples are collected at each study visit. Participants who were vaccinated against seasonal flu had an extra sampling visit, two weeks after vaccination. The flu vaccines which were administered during the study period included Vaxigrip Tetra (Sanofi S.A., Gentilly, France) and Influvac Tetra (Abbot Biologicals B.V., Olst, The Netherlands). In addition, study participants presenting with symptoms of an ILI or ARI had two extra sampling visits: one within five days of symptom onset and another two to three weeks after symptom onset during the attenuation of their illness.

Nasopharyngeal swabs (stored in universal transfer medium - UTM^®^: Viral Transport [Copan, California, USA]) were collected to detect an ongoing SARS-CoV-2 infection by RT-qPCR. Serum (8 mL CAT Serum Separator Clot Activator Vacuette^®^ Tubes, Greiner Bio-One, Kremsmünster, Austria) was collected for serological measurements. Heparinised blood (x 10 mL plastic BD Vacutainer Lithium Heparine tubes (BD, New Jersey, USA) was collected for plasma and peripheral blood mono-nuclear cell (PBMC) isolation for cellular immunity studies. Oral fluid (Oracol Saliva System, Malvern Medical Developments Ltd, Worcester, UK) was collected to study mucosal immunity.

### Questionnaires

Participants completed questionnaires at baseline, at the end of the study and in the case an ILI or ARI occurs. Staff members received a personal link to fill out the questionnaires themselves on-line, while residents were assisted by the study nurses.

Detailed information was requested from the participant for future multivariate analyses. This information includes participant age, gender, self-reported weight and height, ethnicity, smoking status, physical activity, staff members profession, current medication use, prior SARS-CoV-2-test results (i.e. RT-qPCR, lung CT-scan, and/or serology), and vaccination status (i.e. pneumococcal vaccination in the past five years and influenza vaccination in the past year).

Participants were required to answer questions regarding any previous COVID-19 episode or influenza-like symptoms. These questions recorded the symptoms, their duration, the impact of COVID-19 on their daily activities, and the severity of the illness as approximated by hospitalisation and/or ventilation requirements.

Standardised questionnaires were also provided and included a standardised measure for the health status: EQ-5D five-level (EQ-5D-5L) by EuroQol [20]. For residents, frailty was assessed via the 9-point Clinical Frailty Scale [21]. In addition for the residents, the quality of daily living was also asked with the Katz scale (i.e. records the independence of activities of daily living [ADL] by grading six activities [22]) and the MMSE score (i.e. a measure of cognitive impairment in older adults based on a 30-point questionnaire [23]).

#### Baseline

The questionnaire with detailed information was filled out by participants, as was the information on previous COVID-19 episodes, and all the standardised questionnaires.

#### End of study

The questionnaire at the end of the study collects detailed information limited to current weight, smoking status and physical activity of the participants, information on COVID-19 episodes or influenza-like symptoms that occurred during the study period.

Similar to the baseline, questions regarding a COVID-19 during the study were asked, as well as all the standardised questionnaires.

### Biological measurements at baseline

Nasopharyngeal swabs tested for the presence of SARS-CoV-2 RNA with an RT-qPCR targeting the E gene, according to the protocol by Corman et al. [24]. The Ct cut-off for positivity was 40.

The Wantai SARS-CoV-2 Ab ELISA (cat n°WS-1096; Beijing Wantai Biological Pharmacy Enterprise Co. Ltd., China) detects anti-receptor binding domain (RBD) IgG, IgA and IgM simultaneously in serum. *In house* and reported validations show a test specificity of 99.6% and a sensitivity of 100% at 14 days post-clinical illness onset [25–27].

### Statistical analysis

Database management and data analysis were performed with the R software (version 4.0.3.). Descriptive statistics are used to describe the baseline characteristics. Mean ± standard deviation (±SD) is given for continuous variables and the proportion (%) for categorical variables. Normality of data distributions was tested with the Shapiro-Wilk statistics and visually with quantile-quantile (Q-Q) plots.

We used bivariate statistical testing for assessing the proportions of categorical variables (*χ*^2^-statistics) and the number of symptoms (parametric analysis: unpaired t-test; or non-parametric analysis: Mann-Whitney test), comparing the naïve participants with the previously infected.

## Results of baseline characteristics

### Recruitment and sample size

**Fig. 2:**
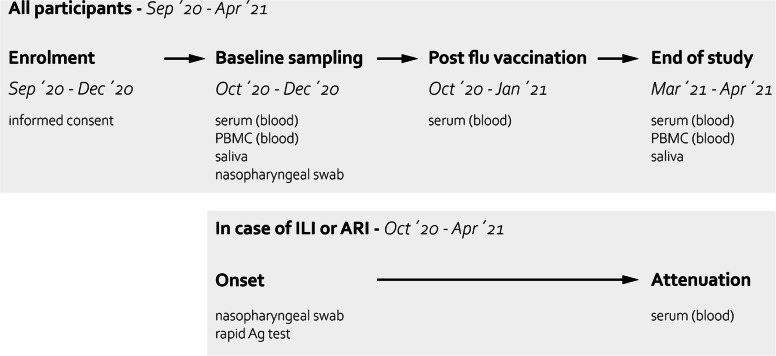
Flow chart of the study population. 1,359 participants of nursing homes provided us with an informed consent. Reasons for withdrawal are provided for the 116 drop-outs. 33 participants did not complete the baseline questionnaire, resulting in a final study population of 1,226 with 374 nursing home residents and 852 staff members

Recruitment of participants started on September 24^th^, 2020, and ended on December 8^th^, 2020. A total of 1,375 participants initially provided informed consent to participate (Fig. 2). 116 individuals (8.4%) withdrew from the study resulting in a final sample size of 1,226 participants. Baseline sampling was completed on December 8^th^, 2020) and sampling after flu vaccination was completed on January 25^th^, 2021).

### Population characteristics

Sociodemographic characteristics of all participants are presented in Table 1. Between March and August 2020, most participants were routinely tested by RT-qPCR with a nasopharyngeal swab (n = 1,197 [97.6%]) and a minority had their blood drawn for a SARS-CoV-2 antibody test (n = 200 [16.3%]). At baseline, 18 participants (1.5%) tested SARS-CoV-2 positive by RT-qPCR. The current study includes 374 residents (30.5%) and 852 staff members (69.5%).

**Table 1:** Sociodemographic characteristics of the total study population (n=1,226), the staff members (n=852), and the residents (n=374). Data is presented as mean (SD) or n (%)

	Resident (n=374)	Staff (n=852)	Total (n=1,226)
Age, years	81.9 (10.7)	44.2 (11.5)	55.7 (20.7)
Weight^1^, kg	70.4 (15.6)	72.9 (14.4)	72.1 (14.9)
Height^2^, cm	166.0 (9.2)	167.0 (8.2)	166.9 (8.5)
BMI^3^, kg/m^2^	25.5 (5.3)	26.1 (4.8)	26.0 (4.9)
Gender			
*Male*	134 (35.8%)	125 (14.7%)	259 (21.1%)
*Female*	240 (64.2%)	727 (85.3%)	967 (78.9%)
Ethnicity			
*European*	371 (99.2%)	747 (87.7%)	1,118 (91.2%)
*Sub-Saharan Africa*	3 (0.8%)	36 (4.2%)	39 (3.2%)
*North-African*	0 (0.0%)	21 (2.5%)	21 (1.7%)
*Asian*	0 (0.0%)	24 (2.8%)	24 (2.0%)
*Latin*	0 (0.0%)	8 (1.0%)	8 (0.7%)
*Mixed*	0 (0.0%)	9 (1.1%)	9 (0.7%)
*Unknown*	0 (0.0%)	7 (0.8%)	7 (0.6%)
Smoke			
*Current smoker*	48 (12.8%)	162 (19.0%)	210 (17.1%)
*Past smoker*	56 (15.0%)	71 (8.3%)	127 (10.4%)
*Non-smoker*	270 (72.2%)	619 (72.7%)	889 (72.5%)
Physical activity per day			
*None*	18 (5.2%)	72 (8.6%)	96 (7.7%)
*Less than 30 minutes*	151 (43.5%)	135 (16.8%)	315 (25.7%)
*30 - 60 minutes*	133 (38.3%)	219 (27.3%)	376 (30.7%)
*At least 60 minutes*	45 (13.0%)	376 (46.9%)	439 (35.8%)

^1^Data available for 368, 846, and 1,220 participants, respectively

^2^Data available for 237, 851, and 1,088 participants, respectively

^3^Data available for 236, 845, and 1,081 participants, respectively

#### Residents

The 374 residents had an average age of 81.9 years (±10.7) and a mean BMI of 25.5 kg/m^2^ (±5.3). More than two-thirds was female (64.4%), and 99.2% was of European descent. Most residents never smoked (72.2%).

#### Staff

Staff members had an average age of 44.2 years (±11.5) and mean BMI of 26.1 kg/m^2^ (±4.8). The majority was female (85.3%) and 747 were of European descent (87.7%). 619 (72.7%) staff members reported to have never smoked. Nurses (23.2%) and nursing assistants (29.7%) were the most included professions (Table 2). In total, 52 (6.1%) staff members worked in specialised COVID departments within their respective nursing homes. 27 of them indicated to have worked solely in these (temporary) departments.

**Table 2:** Profession of the staff members at the nursing home. Data is available for 849 staff members

Profession	Staff (n=849)
Medical	11 (1.3%)
*Medical Doctor*	7 (0.8%)
*Not specified*	4 (0.5%)
*Nurse*	198 (23.2%)
Paramedical	355 (41.7%)
*Nursing assistant*	253 (29.7%)
*Physical therapist*	48 (5.6%)
*Occupational therapist*	37 (4.3%)
*Logopaedics*	3 (0.4%)
*Psychologist*	5 (0.6%)
*Not specified*	9 (1.1%)
Non-medical	258 (30.3%)
*Administration*	87 (10.2%)
*Support staff*	97 (11.4%)
*Kitchen staff*	37 (4.3%)
*Animators*	18 (2.1%)
*Social worker*	2 (0.2%)
*Not specified*	17 (2.0%)
COVID department	27 (3.2%)

### SARS-CoV-2 history at baseline

Previous nasopharyngeal RT-qPCR and serology data indicated that 445 participants reported to have had a positive SARS-CoV-2 RT-qPCR or serology test between March and August 2020, while 781 reported no or only negative RT-qPCR or serology tests (Table 3). Taking serology at baseline into account, 586 participants (47.8%) had neither a positive SARS-CoV-2 RT-qPCR nor a positive anti-RBD IgG antibody test and are therefore categorised as ‘naïve’. The remaining 640 participants (52.2%) are categorised as ’previously infected’. Of the latter group, 61 (9.5%) had a positive RT-qPCR but no detectable antibodies at baseline, 182 (28.4%) never had a positive RT-qPCR but did have detectable antibodies at baseline, and 397 (62.0%) had a previous positive RT-qPCR result and detectable antibodies at baseline.

**Table 3:** Past SARS-CoV-2 infection and seroprevalence at baseline sampling for the total population (n=1,226), staff (n=852), and residents (n=374). The SARS-CoV-2 RT-qPCR results between March and August 2020 were self-reported at enrolment. Seroprevalence was assessed at baseline sampling

Prior RT-qPCR result	Baseline Serology	Resident (n=374)	Staff (n=852)	Total (n=1,226)
Positive	Positive	93 (25.1%)	304 (35.6%)	397 (32.4%)
Positive	Negative	19 (5.1%)	42 (4.9%)	61 (4.9%)
Negative	Positive	76 (20.5%)	106 (12.4%)	182 (14.8%)
Negative	Negative	183 (49.3%)	403 (49.3%)	586 (47.8%)

As expected per our study protocol, the proportion of naïve participants did not differ between residents and staff members (52.4% and 47.4% respectively; p = 0.52). For previously infected participants, fewer residents had detectable Ab after a positive RT-qPCR test than staff members (24.4% and 35.5%, respectively; p = 0.0004). Conversely, more residents than staff members had detectable Ab without a previous positive RT-qPCR (24.4% and 14.2%, respectively; p = 0.0004). The proportion of participants with a previous positive RT-qPCR and no detectable Ab at baseline did not differ between residents and staff members (2.1% and 4.6%, respectively; p = 0.99).

### Symptoms from March to August 2020

**Fig. 3:**
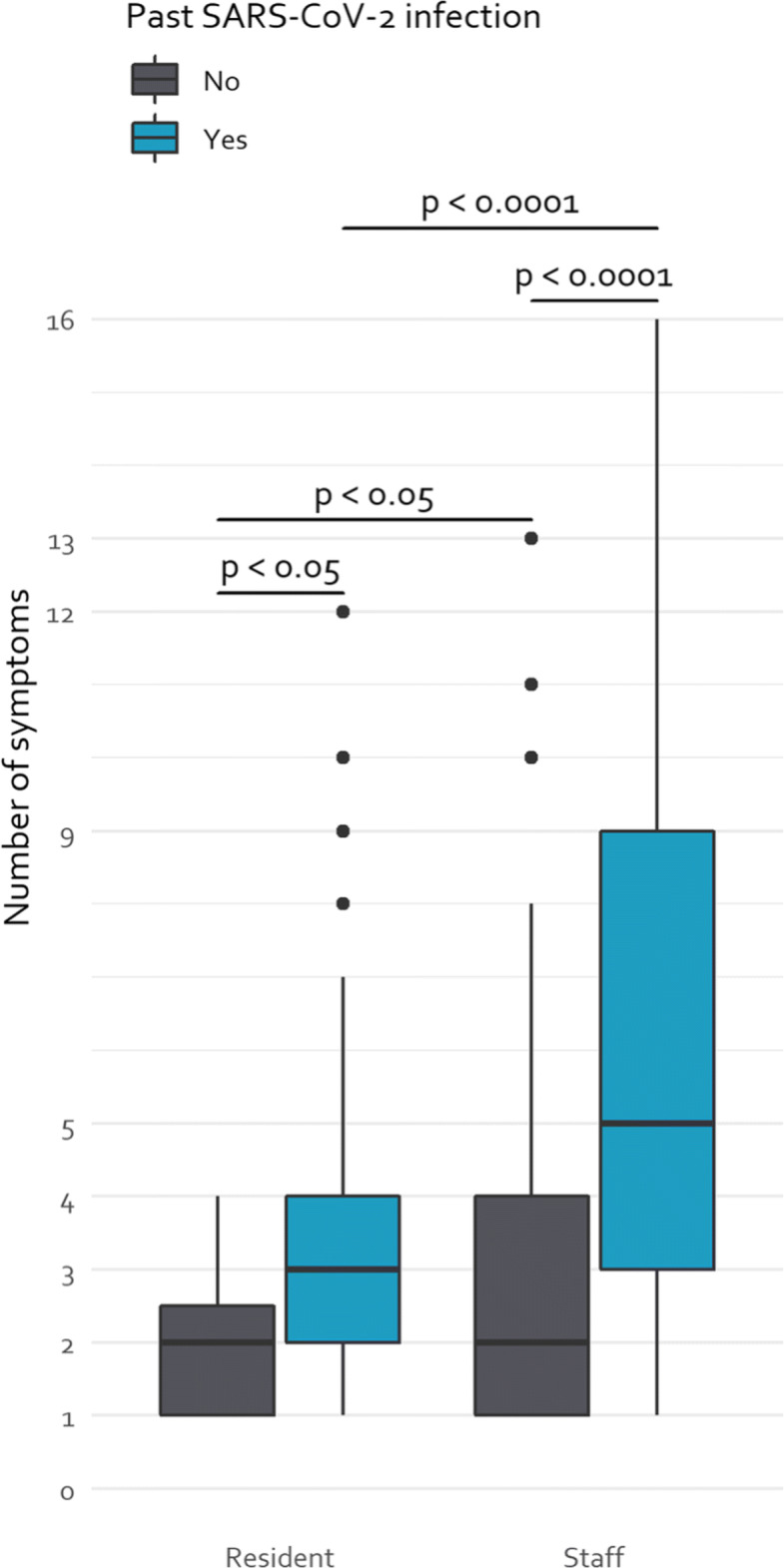
Number of symptoms in staff members and residents of nursing homes. Residents reported an average of 3.1 (±2.4) symptoms during a past SARS-CoV-2 infection, compared to 1.9 (±1.1) symptoms for residents without a previous infection. For staff members with a past SARS-CoV-2 infection, the number of symptoms averaged at 6.1 (±4.0) while those without an infection averaged 3.1 (±2.2) symptoms

Previously infected participants reported significantly more symptoms than naïve participants (p < 0.0001), at 5.5 (±3.9) and 3.0 (±2.3) symptoms, respectively (Fig. 3). This effect was the strongest in the staff members, as those with a past infection had 6.1 (±4.0) symptoms compared to 3.1 (±2.2) symptoms for naive staff members (p <0.0001). In residents, a similar effect was observed as the number of symptoms during a past SARS-CoV-2 infection was higher compared to the naïve residents (3.1 vs 1.9 symptoms, p = 0.0155).

#### Residents

From March until August 2020, 188 residents had a previous infection, of whom the most (111 [59.0%]) reported to have had no symptoms during their prior infection (Table 4). Fatigue (40 [21.3%]) and fever (39 [20.7%]) were the most and second most reported symptoms, respectively.

**Table 4:** Self-reported symptoms the 640 ’past SARS-CoV-2 infection’ participants experienced from March 1^st^ to August 31^st^ 2020 during their COVID-19 episode. Chi-squared statistics (*χ*^2^) was used to compare the residents with the staff members

Symptom	Resident (n=188)	Staff (n=452)	Total (n=640)	*χ*^2^*p*-value
No symptoms	111 (59%)	127 (28.1%)	238 (37.2%)	<0.0001
Fatigue	40 (21.3%)	256 (56.6%)	296 (46.2%)	<0.0001
Ageusia and/or anosmia	12 (6.4%)	202 (44.7%)	214 (33.4%)	<0.0001
Headache	7 (3.7%)	195 (43.1%)	202 (31.6%)	<0.0001
Cough	34 (18.1%)	149 (33.0%)	183 (28.6%)	<0.0001
Dyspnea	30 (16.0%)	148 (32.7%)	178 (27.8%)	<0.0001
Fever	39 (20.7%)	141 (31.2%)	180 (28.1%)	0.0098
Pain (any)	11 (5.9%)	120 (26.5%)	131 (20.5%)	<0.0001
*Muscle pain*	5 (2.7%)	99 (21.9%)	104 (16.2%)	<0.0001
*Joint pain*	2 (1.1%)	76 (16.8%)	78 (12.2%)	<0.0001
*Chest pain*	2 (1.1%)	53 (11.7%)	55 (8.6%)	<0.0001
*Abdominal pain*	1 (0.5%)	16 (3.5%)	17 (2.7%)	0.06
Shivering	4 (2.1%)	118 (26.1%)	122 (19.1%)	<0.0001
Sore throat	5 (2.7%)	116 (25.7%)	121 (18.9%)	<0.0001
Rhinitis	7 (3.7%)	87 (19.2%)	94 (14.7%)	<0.0001
Diarrhoea	13 (6.9%)	70 (15.5%)	83 (13.0%)	0.0049
Nausea	7 (3.7%)	46 (10.2%)	53 (8.3%)	0.0111
Confused	7 (3.7%)	23 (5.1%)	30 (4.7%)	0.59
Anorexia	6 (3.2%)	22 (4.9%)	28 (4.4%)	0.46
Worsening prior respiratory problems	4 (2.1%)	11 (2.4%)	15 (2.3%)	1.00
Rash	0 (0.0%)	10 (2.2%)	10 (1.6%)	0.09

For the 183 naïve residents, 161 reported to have had no symptoms (88.0%; Table 5).

**Table 5:** Self-reported symptoms the 586 ’no past SARS-CoV-2 infection’ participants experienced March 1^st^ to August 31^st^ 2020. Chi-squared statistics (*χ*^2^) was used to compare the residents with the staff members

Symptom	Resident (n=183)	Staff (n=403)	Total (n=586)	*χ*^2^*p*-value
No symptoms	161 (88.0%)	291 (72.2%)	452 (77.1%)	<0.0001
Fatigue	4 (2.2%)	50 (12.4%)	54 (9.2%)	<0.0001
Ageusia and/or anosmia	0 (0.0%)	3 (0.7%)	3 (0.5%)	0.59
Headache	1 (0.5%)	56 (13.9%)	57 (9.7%)	<0.0001
Dyspnea	3 (1.6%)	21 (5.2%)	24 (4.1%)	0.07
Cough	7 (3.8%)	30 (7.4%)	37 (6.3%)	0.14
Fever	2 (1.1%)	17 (4.2%)	19 (3.2%)	0.08
Pain (any)	2 (1.1%)	5 (1.2%)	7 (1.2%)	1.00
Muscle pain	1 (0.5%)	4 (1.0%)	5 (0.9%)	0.95
Joint pain	1 (0.5%)	4 (1%)	5 (0.9%)	0.95
Chest pain	0 (0.0%)	2 (0.5%)	2 (0.3%)	0.85
Abdominal pain	0 (0.0%)	1 (0.2%)	1 (0.2%)	1.00
Shivering	0 (0.0%)	21 (5.2%)	21 (3.6%)	0.0037
Sore throat	5 (2.7%)	52 (12.9%)	57 (9.7%)	0.0002
Diarrhoea	4 (2.2%)	22 (5.5%)	26 (4.4%)	0.12
Rhinitis	4 (2.2%)	38 (9.4%)	42 (7.2%)	0.0029
Nausea	1 (0.5%)	11 (2.7%)	12 (2.0%)	0.16
Anorexia	1 (0.5%)	0 (0.0%)	1 (0.2%)	0.69
Confused	0 (0.0%)	4 (1.0%)	4 (0.7%)	0.42
Worsening prior respiratory problems	0 (0.0%)	2 (0.5%)	2 (0.3%)	0.85
Rash	0 (0.0%)	5 (1.2%)	5 (0.9%)	0.30

#### Staff

Of the 452 previously infected staff members, the majority indicated to have had fatigue (256 [56.6%]; Table 4). Nearly half of the staff reported symptoms of ageusia and/or anosmia (202 [44.7%]) or a headache (195 [43.1%]). 127 (28.1%) staff members indicated to have had no symptoms during their infection.

291 of the 403 (72.2%) naïve staff members reported to have had no symptoms (Table 5). Only a few reported to have had a headache (56 [13.9%]), a sore throat (56 [12.9%]), or fatigue (50 [12.4%]).

### Persisting symptoms at baseline sampling

Previously infected participants were asked whether they experienced any more symptoms at baseline sampling (approximately one to ten months after infection; Table 6). Only 8 (4.3%) of the 188 previously infected residents indicated to have symptoms at baseline sampling, such as coughing (4 [50.0%]), fatigue and dyspnea (2 [25.0%]). Of the previously infected staff members, 86 (19.2%) indicated to have symptoms at baseline. Most indicated to have fatigue (48 [55.8%]), ageusia and/or anosmia (34 [39.5%]), or dyspnea (31 [36.0%]).

**Table 6:** Persisting self-reported symptoms of the 94 ’past SARS-CoV-2 infection’ participants experienced at baseline sampling. Chi-squared statistics (*χ*^2^) was used to compare the residents with the staff members

Symptom	Resident (n=8)	Staff (n=86)	Total (n=94)	*χ*^2^*p*-value
Fatigue	2 (25.0%)	48 (55.8%)	50 (53.2%)	<0.0001
Ageusia and/or anosmia	1 (12.5%)	34 (39.5%)	35 (37.2%)	<0.0001
Headache	0 (0.0%)	13 (15.1%)	13 (13.8%)	<0.0001
Dyspnea	2 (25.0%)	31 (36.0%)	33 (35.1%)	<0.0001
Cough	4 (50.0%)	4 (4.7%)	8 (8.5%)	<0.0001
Pain (any)	0 (0.0%)	11 (12.8%)	11 (11.7%)	<0.0001
Sore throat	0 (0.0%)	6 (7.0%)	6 (6.4%)	<0.0001
Diarrhoea	0 (0.0%)	2 (2.3%)	2 (2.1%)	0.16
Rhinitis	0 (0.0%)	3 (3.5%)	3 (3.2%)	0.046
Nausea	0 (0.0%)	1 (1.2%)	1 (1.1%)	0.23
Anorexia	1 (12.5%)	1 (1.2%)	2 (2.1%)	<0.0001
Confused	0 (0.0%)	8 (9.3%)	8 (8.5%)	<0.0001
Worsening prior respiratory problems	0 (0.0%)	5 (5.8%)	5 (5.3%)	0.025
Rash	0 (0.0%)	2 (2.3%)	2 (2.1%)	0.16

## Discussion

The PICOV study is a prospective multi-centre study designed to investigate the effects of a prior SARS-CoV-2 infection on the occurrence and severity of ILI and ARI among residents and staff members of nursing homes. At the moment of publication, enrolment, baseline sampling, and follow-up sampling after vaccination have been completed. Comparing the symptoms between nursing home residents and staff members showed some interesting differences. On the one hand, residents have a higher proportion of asymptomatic cases (i.e. negative prior RT-qPCR results and detectable Ab at baseline) than staff members. On the other hand, staff members had a higher percentage of prior positive RT-qPCR swabs. Moreover, staff members with a past SARS-CoV-2 infection systematically report more symptoms than either residents with a past infection and staff members without a past infection. In contrast, residents with a past SARS-CoV-2 infection reported slightly more symptoms than naïve residents, albeit statistically not significant.

Fatigue and fever were the most prevalent self-reported symptoms during a previous infection, accounting for over a third of nursing home residents. A recent systematic review by Neumann-Podczaska and colleagues described symptom data of 1,285 older people (aged 60 years and over) from 20 studies [28]. The authors reported that fever and cough were the most common symptoms at 83.6% and 62.7%, respectively. Although fever was the second highest reported symptom among nursing home residents in our study, only 28.1% reported a fever during their COVID-19 episode. In contrast to these earlier findings [28], our nursing home residents reported less coughing (18.1% in PICOV compared to 62.7% by Neumann-Podczaska). The reasons for these differences are not clear, but could firstly be attributed to memory recall errors as this data is collected retrospectively [29]. Secondly, the study population is different, as our data includes only current residents at nursing homes that survived their SARS-CoV-2 infection. This is in contrast with the systematic review [28], where half of the participants were hospitalised and a fifth of the total population died. Finally, our findings are more in line with findings from care homes in the UK [30], as the authors report a more comparable proportion of fever and coughing.

More than half (56.6%) of the previously infected nursing home staff members reported to be fatigued during their COVID-19 episode, which makes this the most prevalent symptom. This incidence is in line with studies in Italian (47.6%) and Swedish (65.0%) healthcare workers (HCW) in hospitals [31, 32]. The prevalence of most of our symptoms are consistent with the observations made by Rudberg et al. [32]. Moreover, while a quarter of the HCW in Sweden [32] and nearly a third of the Italian HCW [31] documented dyspnoea as a symptom, 27.8% of our staff members mentioned dyspnoea during their COVID-19 illness. Although the prevalence of symptoms in the present cohort is comparable to those observed in HCW, we should note some key differences between these populations. First and foremost, both the Swedish and Italian study investigated HCW in hospitals, which is fundamentally different from a nursing home setting. Moreover, we enrolled fewer nurses and physicians and more nonmedical support staff such as administrative, support, and kitchen staff.

In the PICOV cohort, asymptomatic SARS-CoV-2 infections (i.e. received no or negative RT-qPCR nasal swabs and a positive Ab test) occur more frequent in residents than in staff members. This is in line with recent observations by Mori et al. [3], who noted three times as many asymptomatic cases in older people (60 years and over) than in young people (18 - 60 years old) with a confirmed SARS-CoV-2 infection. This reduced symptomatology with age is corroborated by our PICOV symptom data, as previously infected residents systematically reported fewer COVID-19-related symptoms than previously infected staff members. Symptoms that were reported less frequently among the geriatric population as compared to the staff were ageusia and/or anosmia. This is in agreement with previous findings [33, 34] and could be attributed to normal ageing, as taste and smell tend to subside with age [35].

A third of the staff members reported persistent symptoms at baseline, with fatigue, ageusia and/or anosmia, and dyspnoea being the most prevalent, which corroborates previous findings [36, 37]. In contrast, only a tenth of the residents indicated any persistent symptom at baseline. Most of them indicated to have a cough, fatigue, or dyspnoea. In both residents and staff members, fatigue was still present a few months after their SARS-CoV-2 infection. In 2009, a follow-up study of 233 SARS survivors in Hong Kong observed that 40% of the responders had a chronic fatigue problem at their four-year follow-up [38]. Interestingly, the geriatric population reported fewer symptoms during their SARS-CoV-2 infection and fewer persisting symptoms 1-10 months afterwards. In summary, nearly three quarters of the PICOV participants indicated to have no more nor additional symptoms one to ten months after symptom onset, which is in line with other non-hospitalised COVID-19 individuals [39].

Our study has several strengths. Firstly, we recruited a representative sample of both staff members and residents of nursing homes across Belgium. As such, our research population includes participants 18 to 102 years old, providing a broader range of ages compared to other published studies. Secondly, the participants are followed closely and sampled frequently in the case of ILI or ARI, allowing us to capture the development of SARS-CoV-2 Ab already early on during an infection. Thirdly, additional samples are collected at the various sampling moments, which allows for additional measurements for immune response monitoring.

We also acknowledge a number of limitations. First of all, symptoms of a previous COVID-19 episode were registered one to ten months after symptom onset and are therefore subject to recall bias [40]. Nevertheless the authors proposed that patients tend to recall symptoms better than health-related quality of life. In addition, our case definition of SARS-CoV-2 infection did not allow us to capture those participants who experienced an asymptomatic SARS-CoV-2 infection. Finally, only about one in eight nursing homes which were invited also accepted to participate in the study. This may have introduced a selection bias towards nursing homes which were less affected by the COVID-19 pandemic, possibly because such nursing homes had more human resource and organizational capacity to support participation in a study of this kind.

## Conclusion

Nursing home residents (both infection naïve and previously infected) systematically reported fewer COVID-19-related symptoms than staff members in the period of March to August 2020. Moreover, results from prior nasopharyngeal RT-qPCR and baseline serology show that antibody development after a SARS-CoV-2 infection differs between residents and staff members. As such, we can postulate that disease development and COVID-19-related symptoms are different between a geriatric and younger population. Therefore, the influence of a prior SARS-CoV-2 infection on the occurrence and severity of a future ILI and/or ARI might differ between residents and staff.

## Data Availability

The data sets analysed during the current study are available from the corresponding author on reasonable request.

## Abbreviations

ARI: acute respiratory tract infection
EQ-5D-5L: EQ-5D five-level
HCW: healthcare workers
ILI: influenza-like illness
MMSE: mini-mental state examination
PBMC: peripheral blood mono-nuclear cell
PICOV: prior infection with SARS-CoV-2
RBD: receptor binding domain
RDT: rapid diagnostic test
SARS-CoV-2: severe acute respiratory syndrome coronavirus 2
